# DNA transfer: informed judgment or mere guesswork?

**DOI:** 10.3389/fgene.2013.00300

**Published:** 2013-12-25

**Authors:** Christophe Champod

**Affiliations:** Faculty of Law and Criminal Sciences, School of Criminal Justice, Forensic Science Institute, University of LausanneLausanne-Dorigny, Switzerland

**Keywords:** contact DNA, evaluation, forensic science, weight of evidence, expert testimony

With the advances of analytical sensitivity, it is now possible to detect a DNA profile from minute quantity of DNA. It opens new investigative avenues (in cold cases for example), but also new interpretative challenges. Here, forensic scientists deal with items bearing DNA cellular material from areas showing no visible stain and have limited means to identify the nature of the body fluid involved. Such DNA cells can be considered as trace evidence that can be exchanged for reasons connected to the alleged facts under investigation (generally a direct transfer) but also following alternative and versatile ways (through secondary or tertiary transfer) that have no connection to the facts under investigation. The trace becomes an ubiquitous material that can be found for unconnected reasons. In addition, and especially with trace quantities of DNA, the debate in court is less focused on the issue of the source of the DNA (often the parties will not dispute it), but on the mechanisms whereby the biological material has been transferred (Taroni et al., 2013). In other words, the well-known territory of source level DNA statistics (see Buckleton et al., 2005 for example) does not help with the interpretation process, but the forensic scientist is invited to assess how likely it would be to observe this amount of DNA given various transfer mechanisms. The review by Meakin and Jamieson (2013) led them to conclude that the quantity of DNA or the quality of the profile cannot be used “to reliably infer the mode of transfer by which the DNA came to be on the surface of interest.”

This rather complicated new landscape leads to two questions:

1. Is it the role of the scientist to offer guidance as to the probability of the DNA findings given various transfer mechanisms put forward by the parties depending on the case circumstances?
2. Can a forensic scientist robustly assess the probability of the DNA findings given alleged transfer scenarios in the current state of knowledge?

Regarding the first, my view is that it is definitely the role of the forensic scientist to provide as much guidance to the trier of facts if the knowledge he/she may bring is outside the general knowledge of the court and relevant to the task at hand. Shying away from this duty on the ground that considerations regarding transfer of trace DNA is less known than source level DNA statistics is not acceptable. There is a risk with leaving the presence of DNA to be assessed by others, left to advocacy, when the scientist can bring decisive knowledge (let alone the papers reviewed by Meakin and Jamieson), including highlighting how complex the task may be. We want to avoid the simplistic line of argument that I have heard at times: “We have found DNA corresponding to the defendant on the trigger of firearm, hence he manipulated the gun.” It is crucial for a fair administration of justice that forensic scientists weigh their expectations of the amount of DNA recovered given both views. Hence scientists' guidance is required when the consideration of transfer mechanisms, persistence and background levels of the material has a significant impact on the understanding of the alleged activities and requires expert knowledge. But to provide guidance, the scientist will need information regarding the alleged case circumstances from both prosecution and defense's perspective. The duty may also require the scientist to highlight how little is known on transfer mechanisms and urge for a very careful assessment of the evidential contribution of the forensic findings, regardless of their strength with regards to the issue of the source itself. The absence of knowledge should not be an excuse for a guilty silence and for delegating the task to the fact finder without making explicit the complexity surrounding such an assessment.

In relation to the second question, Risinger (2013) warns against the “abuse of the notion of subjective probability,” … “by simply making their best guess from experience when more should be required.” In contrast, courts (I will concentrate here on the jurisdiction of England and Wales) have recently given a lot of freedom or authority to DNA scientists to exercise their professional judgement even when limited or no published data were available. In R v Reed and Reed (2009), the court ruled that in the context of the analysis of minute quantity of DNA, a reporting scientist is fully entitled to assess and weigh the relative merits of the possible mechanisms whereby cellular material can be exchanged. In that case the forensic scientist testified that, in her experience, it was highly unlikely that the appellants had innocently touched the knives and it was unrealistic that each appellant had passed their DNA to someone else who then transferred it to the pieces of plastic which were found at the victim's address. The court while recognizing that the scientific knowledge on transferability was incomplete, ruled that enough reliability had been demonstrated when the scientist is asked to consider cases where more than 200 picograms of DNA had been recovered. The court however stressed that “care must be taken to guard against the dangers of that evaluation being tainted with the verisimilitude of scientific certainty.” The scientist is then authorized to comment on the probability of the forensic results given various transfer mechanisms as long as he/she makes it clear that we are dealing here with large uncertainty. This judgement led to a few commentaries. Jamieson (2011) highlighted the limited body of evidence represented by the few papers quoted by the court to support their opinion and warned against the view that the personal experience might override scientific research. A worry echoed in an editorial (Nic Daéid, 2010) in *Science and Justice* following the next case against Weller.

In R v Weller (2010), (a case involving the transfer of a reasonable quantity of DNA under the fingernails of the defendant), the defense appealed on the ground that knowledge regarding transfer and persistence mechanisms of DNA was not sufficient for experts to have been able to express an evaluation of the relative merit of the alleged activities. The Court of Appeal confirmed the positions taken in *Reed and Reed*. Given the difficulties of conducting systematically experiments replicating the circumstances in a particular case, the court recognized that a scientist is fully entitled to express a professional opinion on his/her expectation of DNA quantities given each mechanism envisaged by the court if the scientist has sufficient casework day-to-day experience. Jamieson and Meakin (2010) expressed their concerns after *Weller* seeing courts in England and Wales putting more trust in claimed experience than in published, peer-reviewed, publications. Rudin and Inman (2010a) also insisted on the fact that bald experience is not an acceptable substitute for experimental data.

Following these two cases, the Court of Appeal confirmed that view in subsequent rulings, not only in relation to consideration of DNA transfer but also of the sources of complex DNA mixtures. In R v Thomas (2011), a DNA scientist invoked her 12 year experience (and some unpublished and undisclosed data) to suggest that in a three-person DNA mixture, there was a low expectation of finding components matching all those of the appellant adventitiously. In R v Dlugosz et al. (2013), the court, recognizing their extensive professional experience, allowed two DNA scientists to qualify the occurrence of alleles in a complex mixture corresponding to the defendant as a “rare” for one and “somewhat unusual” for the other. The qualitative opinion expressed by the scientists was offered as an acceptable substitute in cases where the mixture is too complex for a quantitative assessment. However, as pointed out by Evett and Pope (2013), “there is no scientific basis for this belief—no scientific literature provides a reliable methodology, scientists are not trained to make such assessments and there is no body of standards to support them. Casework experience is not a substitute.” One needs to assess the robustness of such qualitative opinion through a structured program of proficiency tests: it should not be based on casework data, but on DNA mixtures obtained under controlled conditions. Expressing qualitative judgments on the basis (or assumptions) of casework samples, without any calibration mechanism, is dangerous in my view. The expressed opinion could be the expression of nothing more than the *ipse dixit* of the expert.

The Court of Appeal endorsed such a *laissez faire* approach drawing from a much larger jurisprudence applicable to expertise in general, with some decisions relating to other areas of forensic disciplines. For example, in R v Otway (2011), involving gait analysis, and two other cases, namely R v Atkins and Atkins (2009), (face recognition) and R v T (2010), (footwear mark), the court recognized that an expert may express a qualitative opinion in the absence of quantitative (or statistical) supporting data as long as the subjective nature of the opinion and its foundation are transparently presented without giving more scientific weight to the judgment than it disserves. Edmond et al. (2010) remains rightly skeptical with the approach of dressing an opinion with all the concessions of limitations, but still allowing it, when the real significance of the forensic findings remains simply unknown.

In my view, what is critical, when it comes to offer expert opinions (in the present discussion regarding DNA transfer), is striking the appropriate balance between structured documented data (published or not) and unfettered personal opinion. Should these opinions be based *in extenso* on experience? My answer is clearly negative. I believe that experience constitutes a poor substitute to a systematic and structured acquisition of data. Any scientist offering views as to his/her expectations for the forensic findings under given case-related circumstances should be able to put forward documented sets of controlled experiments whose relevancy to the case under dispute can be argued. A further question is how many controlled experiments should be conducted and how close should they be to the alleged circumstances. In my view that question should be approached on a case-by-case basis using the adversarial mechanisms available to the parties. The major improvement here is that all parties can access and challenge the body of knowledge available to the expert proffering an opinion. As Rudin and Inman (2010a) indicated, the problem with experience only based opinions is that it cannot be challenged beyond the sterile opposition between mere opinions. Requiring the disclosure of structured data opens the route to a new type of debate regarding the relative merits of the assessments provided.

We could legitimately ask, as did Rudin and Inman (2010b), whether or not forensic science has gone too far in terms of sensitivity, meaning that the risks associated with the analysis of irrelevant (meaning not associated with the criminal activities under investigation) items are too high. I believe that the problem lies more in the usage made by law enforcement authorities of such sensitive technologies. There are only gains in terms of investigative leads if we take advantage of sensitive techniques, but maybe these methods should be used only in the investigative phase, not as a basis for evidence relied on at trial. Highly sensitive DNA analysis offers extraordinary ways to enhance an investigation through the suggestion of potential named sources (through DNA databases) for the inquiry to consider. I am not calling for limiting such opportunities. However, moving from such investigative information toward elements of evidentiary purposes to be used in court requires very careful attention. It may well be the case that a decisive investigative information will not be brought to court because of the issues discussed above. This is not a failure of forensic science, but simply an appropriate and fair (re-)positioning of the scientific techniques within the criminal justice process.

## Conflict of interest statement

The author and editor declare that while the author Christophe Champod and the editor Alex Biedermann are currently employed by the Université de Lausanne, Institut de police scientifique, Switzerland, there has been no conflict of interest during the review and handling of this manuscript.
